# Could Dopamine Agonists Aid in Drug Development for Anorexia Nervosa?

**DOI:** 10.3389/fnut.2014.00019

**Published:** 2014-11-03

**Authors:** Guido K. W. Frank

**Affiliations:** ^1^Department of Psychiatry, School of Medicine, University of Colorado, Aurora, CO, USA; ^2^Department of Neuroscience, School of Medicine, University of Colorado, Aurora, CO, USA

**Keywords:** anorexia nervosa, dopamine, medication, drug, anxiety, cognitive flexibility, eating

## Abstract

Anorexia nervosa is a severe psychiatric disorder most commonly starting during the teenage-years and associated with food refusal and low body weight. Typically there is a loss of menses, intense fear of gaining weight, and an often delusional quality of altered body perception. Anorexia nervosa is also associated with a pattern of high cognitive rigidity, which may contribute to treatment resistance and relapse. The complex interplay of state and trait biological, psychological, and social factors has complicated identifying neurobiological mechanisms that contribute to the illness. The dopamine D1 and D2 neurotransmitter receptors are involved in motivational aspects of food approach, fear extinction, and cognitive flexibility. They could therefore be important targets to improve core and associated behaviors in anorexia nervosa. Treatment with dopamine antagonists has shown little benefit, and it is possible that antagonists over time increase an already hypersensitive dopamine pathway activity in anorexia nervosa. On the contrary, application of dopamine receptor agonists could reduce circuit responsiveness, facilitate fear extinction, and improve cognitive flexibility in anorexia nervosa, as they may be particularly effective during underweight and low gonadal hormone states. This article provides evidence that the dopamine receptor system could be a key factor in the pathophysiology of anorexia nervosa and dopamine agonists could be helpful in reducing core symptoms of the disorder. This review is a theoretical approach that primarily focuses on dopamine receptor function as this system has been mechanistically better described than other neurotransmitters that are altered in anorexia nervosa. However, those proposed dopamine mechanisms in anorexia nervosa also warrant further study with respect to their interaction with other neurotransmitter systems, such as serotonin pathways.

## Introduction

The eating disorder anorexia nervosa is a severe psychiatric disorder with high mortality. Anorexia nervosa usually begins during adolescence (1) with a lifetime prevalence of about 0.9% of the female and 0.3% of the male population (2). Between 20 and 40% of individuals drop out of treatment and only about 30% of individuals with anorexia nervosa have a successful recovery (3).

The hallmark sign of anorexia nervosa is the restriction of energy intake relative to requirements leading to a significantly low body weight (1). This severe disturbance of adequate food intake suggests potential biological factors that “enable” an individual with anorexia nervosa to severely restrict food intake. Anorexia nervosa’s second diagnostic criterion is an intense fear of gaining weight or becoming fat, even though underweight. This fear may be conditioned after comments from the social environment. The third symptom cluster is the disturbance in the way one’s body weight or shape is experienced, undue influence of body weight or shape on self-evaluation, or denial of the seriousness of the current low body weight. This distortion of one’s body image is particularly difficult to understand and especially in the restricting type of anorexia nervosa this has psychotic quality (4). The lack of menses in anorexia nervosa has been removed from the diagnostic criteria in DSM-5, but typically the disorder is associated with low gonadal hormone levels, which is important as gonadal hormone levels affect brain function and behavior (5–7). Another typical behavior associated with anorexia nervosa is the resistance to change behavior even when recognizing the severe negative consequences the illness causes (8). This suggested that individuals with anorexia nervosa have deficits in cognitive flexibility.

The causes for developing anorexia nervosa are considered complex interactions between psychosocial and neurobiological abnormalities (1). This has limited the development of neuroscience-based treatments (9), and no medication or other biological treatment has been approved for the disorder (10).

For a long time, the brain serotonin system was the primary focus of neurobiological research in anorexia nervosa, but this system is very complex and difficult to model in terms of distinct receptor-stimulus-behavior associations. More recently research suggested that brain dopamine circuitry could be a potential key player in the pathophysiology of altered food intake in anorexia nervosa (9, 11, 12). This is important as the dopamine system has been particularly well characterized, computational models exist that predict dopamine neuron activation, and this system can be manipulated pharmacologically (13, 14).

This article will review research on dopamine in anorexia nervosa and then provide evidence that brain dopamine function is tied to a variety of core and associated symptoms of anorexia nervosa. Targeting this system pharmacologically especially with dopamine agonists could be a promising approach to ameliorate this illness and facilitate recovery.

## Studies on Brain Dopamine in Anorexia Nervosa

The main dopamine releasing neurons arise from the midbrain ventral tegmental area and substantia nigra and project to caudate and putamen, the medial prefrontal, cingulate, and entorhinal cortex (mesocortical projections), as well as to the limbic system with nucleus accumbens, amygdala, septum, and piriform cortex (mesolimbic projections) (15). The best-studied receptors of this system are the G-protein coupled dopamine D1 and D2 receptors, but other receptor types have also been identified (15). The human dopamine D1 receptor is a postsynaptic receptor that mediates more directly behavior and the D2 receptor is a presynaptic auto-receptor that regulates dopamine release in a negative feedback fashion; D1 receptors activate, whereas dopamine D2 receptors decrease adenylyl-cyclase activity, and both receptor types are distributed throughout the cortex, subcortical nuclei, and brain stem (16). Importantly, continuous exposure to dopamine antagonists increases dopamine receptor binding sites, especially of the dopamine D2 receptor, and prolonged stimulation with dopamine agonists desensitizes and reduces the number of dopamine receptors (15–17).

A variety of studies indicated altered dopamine function in anorexia nervosa. In a group ill with anorexia nervosa, cerebrospinal fluid homovanillic acid, the major dopamine metabolite, was reduced by about 30% compared to controls (18), and recovered restricting type anorexia nervosa subjects had significantly reduced cerebrospinal fluid homovanillic acid concentrations compared to controls (19). In a positron emission tomography study, a mixed group of recovered restricting type and recovered binge-eating/purging-type anorexia nervosa women had increased dopamine D2/D3 receptor binding in the antero-ventral striatum (20). This suggested that those receptors might be up-regulated in response to low intrinsic dopamine levels, and such alterations could be a trait vulnerability or develop over time. A limitation here is that positron emission tomography studies test available dopamine receptor profiles, but they cannot test well the functionality of those receptors in relation to actual behavior. Others found increased eye-blink in anorexia nervosa individuals compared to controls (21), which suggested increased central dopamine activity (22). In summary, those studies suggest that neuronal or synaptic dopamine may be reduced, but that dopamine receptors could be increased in number or sensitivity in a compensatory or negative feedback fashion (22). This dynamic could provide clues that a down-regulation of receptor sensitivity might be an important therapeutic goal, despite or maybe even consistent with the notion of low extracellular dopamine in anorexia nervosa.

## Brain Dopamine, Reward, and the Regulation Food Intake

Food intake is driven by a complex interplay between cognitive, emotional, and energy homeostasis maintaining mechanisms between brain and body (23). This process has been distinguished in a cognitive or cephalic phase that involves desire or craving, as well as a consummatory phase involving the hedonic experience. These mechanisms were then further described as the dopamine function associated “wanting” or the drive to approach a reward, and “liking” or the hedonic experience during food consumption associated with opioid system activity (24, 25). The overarching circuitry here is the brain reward system, which integrates more basic metabolic hunger signals with higher order processing of taste and cognitive–emotional factors that regulate food approach and eating (26). Important brain regions that regulate those processes include: (1) the insula as the primary taste cortex and central gateway to (2) the dopaminergic basal ganglia and midbrain, to (3) higher order brain centers including the prefrontal and (4) the cingulate cortex that integrates cognition and emotions, (5) the orbitofrontal cortex, which determines when to stop eating a type of food, and (6) the amygdala that associate stimuli with emotional experience and that are thought to modulate dopamine circuitry in midbrain and striatum (27–30).

The dopamine neurotransmitter system plays a central role in the motivational aspects of food approach (25, 31, 32). Dopamine neurons respond to novel as well as unexpected stimuli, and provide a learning signal to “stamp in response–reward and stimulus–reward associations” to control motivated behavior (31). Recent research also suggests that the dopamine signal within the reward circuitry is a composite of quality, quantity, and effort to acquire the reward (26). Especially important for the study of anorexia nervosa is research that suggests that extremes of eating behavior can modulate the dopamine system. Animal studies suggested heightened brain reward response after food restriction, including increased ventral striatal dopamine release or lower lever press threshold to receive a reward stimulus (33, 34). On the contrary, excessive food *intake* has been associated with reduced caudate dopamine receptor availability in rodents, after they were fed with a high caloric “cafeteria food” diet (35). Those results strengthen the notion that extremes of food intake might be associated with opposite dopamine system alterations.

Also important is that food restriction affects reward sensitivity in adolescence differently than in adults as anorexia nervosa typically has its onset during teenage-years. For instance, rodent studies showed higher motivation and activity after food restriction in adolescent animals compared to adults (36). Human brain imaging studies showed both hypo- and hyper-activation of reward circuits in adolescents (37), but with transition from adolescence to adulthood striatal activation seems to increase in response to highly salient stimuli (38).

Those age specific observations could have important implications, as dopamine circuitry might be particularly vulnerable to severe food restriction or other extremes of eating behaviors during adolescence. In support of the notion that adolescence is a critical period for dopamine receptor development is that these and other monoamine receptors reach adult levels during adolescent years (39). Disruptions in this maturational process could promote specific receptor dysfunction as well as widespread alterations in proliferation, migration, and differentiation of normal cortical and striatal neurocircuitry (40). Such developmental interferences during adolescence could similarly have implications for other disorders such as schizophrenia, which typically has its onset around age 16 and has been associated with dopamine function (41).

## Taste Reward Processing in Anorexia Nervosa

Taste is an important determinant of food intake (42) and several studies investigated brain taste reward circuits in anorexia nervosa. Recovered anorexia nervosa individuals showed reduced functional brain response to *repeated* but increased response to *randomly* given taste reward stimuli (43, 44), and those results in opposite directions suggest that unpredictable versus predictable stimulus presentation may be important when studying anorexia nervosa. The question remains, whether findings in recovered anorexia nervosa are predating the disorder as potential traits or are effects of the illness. Another approach is to pair unconditioned taste stimuli with conditioned visual or auditory stimuli and then at times omit an expected taste delivery or deliver a taste stimulus when none was expected. This leads to a discrepancy between reward anticipated or predicted and the reward actually received, the so called prediction error, which is reflected in dopamine neuronal response (45). Brain dopamine neurons respond with a phasic burst to unexpected salient or reward stimuli, but a dip in tonic neuronal activity when an expected reward stimulus is not received (46). The presynaptic dopamine D2 receptor has been associated with the response to unexpected stimulus omission, while the postsynaptic dopamine D1 receptor is thought to mediate response to unexpected reward stimulus receipt (14).

We have previously applied a prediction error taste reward task using sugar solution and visual conditioned cues in anorexia nervosa and compared this group with obese individuals. We wanted to answer the question whether we could detect neurobiological alterations that lie on opposite ends (47), as suggested by the above-described animal studies (33, 35). We found that insula and ventral striatum prediction error response were greater or more sensitive in anorexia nervosa compared to controls, while obese individuals showed reduced response, supporting the notion that BMI and extremes of food intake are directly related to prediction error and thus dopamine brain response in humans. Those alterations could be due to altered dopamine receptor function. Research in non-clinical human samples supported this hypothesis, as acute application of the dopamine reuptake inhibitor amphetamine and the dopamine D2 antagonist haloperidol could manipulate prediction error related brain response (48), and the dopamine D2 receptor density regulating TaqIA A1 gene variant determined brain response to food stimuli in the midbrain and orbitofrontal cortex (49).

This suggested dopamine hyper-sensitivity in anorexia nervosa is not meant to imply that the so called “reward stimuli” (such as sugar solution) used in reinforcement learning paradigms are necessarily a reward in the sense of positive reinforcer or pleasant experience for individuals with anorexia nervosa. Rather brain dopamine circuits in anorexia nervosa could be hypersensitive to salient stimuli in general. Salient stimuli (conditioned and unconditioned) activate brain dopamine circuits (50), and highly elevated sensitivity to salient rewarding or punishing stimuli (51) in anorexia nervosa could be related to elevated dopamine system activity. Such high sensitivity to salient stimuli could be a trait, exaggerated when underweight and contributing to intolerance of uncertainty (52).

One might argue that if the dopamine system is hypersensitive in anorexia nervosa, it should promote food seeking instead of avoidance. There is little research that investigated those phenomena in humans, but starvation in non-eating disordered healthy individuals lead to difficulties with the modulation of how much food to eat (53), a phenomenon commonly seen in AN when restoring weight and not uncommonly resulting in a period of binge-eating (54). Such dysregulated eating behavior could be related to altered dopamine receptor and reward system sensitivity (33, 34). What makes the pathophysiology of AN unique and different compared to any animal model are psychological factors such as the extreme fear of weight gain, which collides with the neurobiological drive to eat, which may create a severe internal conflict and leading to the typical clinical picture of anorexia nervosa. High trait anxiety, maybe related to serotonin system alterations (55), may underlie the extreme fear focused on weight gain, and the dopamine system related drive to eat may not be strong enough to overcome anxiety that is driven by an opposing serotonin system function (56).

## Fear of Weight Gain and Learned Behavior

Previous research has shown that anorexia nervosa is associated with high premorbid and comorbid anxiety disorders (2). Typical anxiolytic medications such as benzodiazepines have not helped decrease core features of anorexia nervosa (10). Anxious traits may make individuals more prone to respond to anxiety provoking cues from the environment, and the preoccupation with the fear of being fat could be a particular vulnerability for developing an eating disorder such anorexia nervosa. High comorbid depression may also aid in this dynamic (2). The spectrum of anxiety disorders is very broad, but what these disorders share is an attention bias toward threatening stimuli (57). That is, when an individual with anxious traits becomes focused on an anxiety-inducing topic, such as to worry about their weight and shape after comments from the environment she may not be able to let go but keeps focusing on it. (58). One direction of research suggested that such selective attention to negative stimuli be related to less effective serotonin transporter genotype alleles (59). However, serotonin reuptake inhibitors did not help in the treatment of the core features of anorexia nervosa including the attention bias toward their own body. Other recent research now finds evidence that attention bias is modulated by polymorphisms in monoamine oxidase and dopamine beta-hydroxylase genes (60). Others found that depletion of the dopamine precursors phenylalanine and tyrosine resulted in a bias toward immediate monetary reward selection (as opposed to reward delay) in individuals with the catechol-*O*-methyltransferase val/val genotype, a genotype that has been associated with lower dopamine tone in the frontal cortex (61). This suggested that dopamine activity may guide bias toward and processing of salient and potentially anxiety provoking stimuli and alterations could contribute to food avoidance.

An increasing body of literature is now describing how dopamine neuron activation across the amygdala, ventral tegmental area, striatum, and hippocampus is important for fear extinction acquisition, whereas fear extinction consolidation requires prefrontal cortical dopamine D1 and D2 receptor stimulation (58, 62, 63). Particularly important in this context are the effects of low gonadal hormones in anorexia nervosa. Psychotherapy of anorexia nervosa is designed to reinstate normal eating behavior and extinguish fears of getting fat by exposure to food stimuli and eating (64). Research indicates that there is an interaction between hormonal state and fear extinction, and especially females in a low-estrogen state may benefit from dopamine receptor stimulation when trying to suppress previous fears after extinction training (“extinction retrieval”) (65). In that study (65), rodents underwent cue (tone) induced fear conditioning (mild foot shock) and then received the dopamine D1 agonist SKF38393 or a sham injection (vehicle) before extinction learning. During the following extinction retrieval phase, the vehicle treated female animals in the low-estrogen phase showed greater anxiety (freezing) compared to the high-estrogen phase females. However, pretreatment with the dopamine D1 agonist reversed this deficit in low-estrogen animals but worsened freezing in high-estrogen females, and there were no drug effects on males (65). Thus dopamine D1 receptor stimulation could support anxiety reduction specifically in females with anorexia nervosa, as the disorder is typically associated with low gonadal hormone levels.

Other studies have implicated the dopamine D2 receptor in conditioned fear response. For instance, the dopamine D2 agonist quinpirol applied to the midbrain ventral tegmental area reduced expression of conditioned fear response in rodents and it was hypothesized that this was mediated by presynaptic dopamine release modulation and specifically via the dopamine D2 receptor, as dopamine D1 stimulation did not produce this effect (66). However, regional specific blockade of the dopamine D2 receptor in the amygdala also reduced fear response and it appears that a balance between stimulation and activation of the dopamine D2 receptor within the mesolimbic pathway is needed to reduce fear expression (66). In another study, the D2 receptor agonist quinpirol reduced amygdala dopamine levels and associated fear response. That study further implicated mesolimbic terminals between the midbrain and amygdala in fear response (67). An additional interesting effect of the dopamine D2 agonist quinpirol was to block conditioned fear memories, which affected both fear conditioning as well as extinction (68).

Taken together, the dopamine D1 and D2 receptors appear to be potential targets for treatment of anxiety and modulation of conditioned fear. Receptor stimulation could be promising although systemic application of dopamine D2 blockade facilitated fear extinction as well (69). Most studies generally used acute, short-term designs though. Chronic dopamine D2 receptor antagonist application rather enhances this receptor system over time, while chronic agonists decrease dopamine receptor activity (15, 17). Thus, the effects of dopamine D1 and D2 agonists and antagonists have to be studied over longer periods and in relation to weight state. The dopamine D2 receptor partial agonist aripiprazole showed anxiolytic effects during a fear conditioning paradigm in animals (70) and reduced distress around eating in individuals with anorexia nervosa (71). Those results support the hypothesis that dopamine receptor activation might be particularly beneficial in anorexia nervosa treatment. However, any positive effects are speculative at this point and require careful study.

## Body Image Distortion

One of the most puzzling symptoms in anorexia nervosa is body image distortion, which drives pathologic eating behavior as well as suicide (72). The discrepancy between objective body weight and subjectively perceived “being fat” has already long ago raised the suspicion whether this is a psychotic process and whether antipsychotic medication could be beneficial (73). More recently, an interesting distinction was found. That is, while individuals with both restricting as well as with binge-eating/purging-type anorexia nervosa display this symptom, primarily the anorexia nervosa restrictor type group shows a true psychotic quality (4). The available literature is mixed on potential etiologies of this symptom, but self-perceived hyper-sensitivity to sensory stimuli and altered interoceptive awareness could contribute aside from primary brain dysfunction mediated fixed false believes (74, 75). There are various reasons why dopaminergic substances could be helpful in the treatment of body image distortion. Dopamine receptor stimulation has been shown to decrease the representation of one’s own body parts in the rubber hand illusion paradigm (76), and perhaps an overactive representation of one’s body in anorexia nervosa could be reduced. Second, dopamine D1 and D2 receptors can be found in the human skin (77). That opens the possibility that alterations in the dopamine receptor system in the periphery could also contribute to sending false signals about one’s body size to the brain. While body image distortion has been largely studied in relation to visual perception and conditioned fear, I believe that further investigation of somatic-perceptual alterations in anorexia nervosa could be fruitful. It is uncertain however, whether excessive or inadequate stimulation of dopaminergic pathways contribute to delusional body experiences (4, 78), but those questions warrant further study.

## Cognitive Flexibility

Set-shifting is a neurocognitive concept that refers to the ability to switch between tasks and behaviors flexibly. In other words, set-shifting is the mental ability to change behavior in relation to changing rules and demands. Several studies found that adults with anorexia nervosa have set-shifting deficits in that they tend to perseverate on previously applicable rules (79, 80). Such findings are consistent with the clinical observation that these patients tend to be cognitively rigid and persistent. Reduced set-shifting has also been found in anorexia nervosa individuals who had restored weight as well as unaffected relatives of anorexia nervosa patients (81). Individuals who have maintained long-term recovery from anorexia nervosa (i.e., maintained a stable weight and resumed menses for one year) have also shown set-shifting impairments compared to age-matched healthy controls (82, 83). However, set-shifting in adolescents with anorexia nervosa is normal (84), raising the possibility that set-shifting alterations may become prominent and important for illness prognosis in late adolescence or early adulthood. Given that set-shifting has been associated with brain dopamine function (85) in anorexia nervosa (19, 20), it is possible that set-shifting inefficiencies in anorexia nervosa represent alterations in the dopamine system.

The literature on dopamine receptor manipulation and its effects on set-shifting and cognitive flexibility in humans are small, but available studies suggest that D1 and D2 manipulation affects cognitive flexibility (86). Cognitive flexibility can be described and tested in terms of: (1) reversal learning or the ability to adapt behavior in response to a reversal of reinforcement contingencies, (2) attentional set-shifting or the adaptation of behavior following changes in the relevance of perceptual categories or dimensions, (3) task switching or the rapid switching between stimulus-response sets that have been acquired previously, as well as (4) the ability to behave flexibly in conditions that previously allowed automatic or habitual performance but now the individual has to override this automatism with new behavior (86). Pharmacologic challenge studies indicated that for the regulation of cognitive flexibility a balance between dopamine D1 and D2 receptors in the prefrontal cortex is necessary (85). Importantly, effects of receptor stimulation may vary depending on an individual’s baseline dopamine level. For instance, the dopamine D2 agonist bromocriptine impaired reversal learning in individuals with high, but improved reversal learning in individuals with low dopamine synthesis capacity (87). This could have specific implication for anorexia nervosa, which is thought to be associated with low intrinsic dopamine (18). Further, dopamine D2 stimulation in individuals with low dopamine synthesis capacity improved cognitive flexibility as tested in the Wisconsin Card Sorting Test (88). In another study, dopamine D2 stimulation improved task switching in low dopamine synthesis capacity subjects (89). All in all, there is evidence that dopamine receptor manipulation may aid in improving cognitive flexibility and especially D2 agonists may help with this behavior during a low dopamine state as it is hypothesized for anorexia nervosa.

## Dopaminergic Drugs in the Treatment of Anorexia Nervosa

The above-described research suggests that there are distinct dopamine functions that are associated with feeding, reward processing, body perception, as well as learning and cognitive functions, which suggests that this system may be involved in the pathophysiology of anorexia nervosa.

Various dopaminergic drugs have been shown to affect eating and body weight. For instance stimulants such as the dopamine reuptake inhibitor methylphenidate or the dopamine reuptake inhibiting antidepressant bupropion frequently attenuate food intake and promote weight loss (90). Relevant in the context of the above mentioned temporal difference model studies is that acute application of the stimulant and dopamine reuptake inhibitor amphetamine increased, while the dopamine D2 antagonist haloperidol decreased brain response in a human prediction error paradigm (48), This suggested that modulation of dopamine receptor function could indeed alter brain behavior in anorexia nervosa. That study used a one-time drug application design while chronic application of those agonists or antagonists may have different effects, such as receptor desensitization in response to long-term agonist exposure (16).

Various studies investigated in anorexia nervosa dopaminergic drugs, typically neuroleptics, but the results yielded mixed results at best, and especially controlled studies are rare (91). The medication that was studied most frequently is olanzapine, a dopamine D2 receptor antagonist and an inverse agonist on the serotonin 2A, 2B, and 2C receptors with also antagonistic action on many other receptors, including adrenergic, histaminic, and muscarinic receptor types (15). Two studies in adults with anorexia nervosa found greater weight gain with olanzapine compared to placebo (92, 93), but another study contrasting olanzapine with placebo in adolescents and young adults found not greater weight increase (94) and a study that compared in adults with anorexia nervosa olanzapine plus cognitive behavioral therapy with placebo plus cognitive behavioral therapy did not find any benefits on weight gain from olanzapine (95). A study that compared olanzapine with the antipsychotic chlorpromazine, a strong antagonist on dopamine D1 and D2 as well as an antagonist on serotonin 1A and 2A, adrenergic, muscarinic, and histaminic receptors, did not show benefits from olanzapine on weight gain (96). Another medication, the dopamine D2 and D4 and alpha 1 adrenergic receptor antagonist and serotonin 2A inverse agonist risperidone, studied in adolescents did not show benefits over placebo (97), nor did the dopamine D2, D3, and D4 antagonist pimozide (98), or the relatively selective dopamine D2 and D3 antagonist sulpiride (99). In summary, dopamine antagonists did not prove to be effective in the long run in anorexia nervosa, despite some promising case reports (91, 100).

## Dopamine Receptor Agonists as Potential Pharmacologic Intervention for Anorexia Nervosa

A recent study showed that a dopaminergic challenge using the drug amphetamine lead to anxiety in anorexia nervosa as opposed to euphoria in healthy controls (101). This further supported the above-described hypothesis that the dopamine system is hypersensitive in the disorder. Importantly, as described above, while absolute dopamine levels may be low, there may be an under- and malnutrition associated up-regulation of dopamine receptors (102) and potentially a hyper-sensitivity of reward responsiveness (103). If in fact there is a hyper-sensitivity of the dopamine D1 and D2 receptors in anorexia nervosa then long-term application of dopamine receptor antagonists could further increase receptor availability and system activity (15–17). On the contrary, cautious application of dopamine receptor agonists could be beneficial in anorexia nervosa as it would result in a net decrease in dopamine binding sites and desensitization over time and possibly reduced response sensitivity (15, 104–107). Such dopamine receptor down-regulation then might attenuate reward system responsiveness, aid in habituation to re-feeding and fear extinction especially in females with anorexia nervosa in a low-estrogen state (65), reduce conditioned fear response (66, 68), and reduce cognitive rigidity (86) and body image distortion (4). All in all long-term use of dopamine system antagonistic medications is not supported by the available studies, but whether long-term use of dopamine agonists may in fact down-regulate this system and ameliorate anorexia nervosa core symptoms and outcome warrants specific study.

## Potential Dopamine–Serotonin Interactions in Anorexia Nervosa

This review is primarily focused on dopamine function and its role in anorexia nervosa. However, brain neurotransmitter systems obviously do not act independently, which may have direct impact on eating disorder pathology. For instance, one study found that the interaction between serotonin transporter and dopamine D2/3 receptor binding was related to anxiety in anorexia nervosa (108). Above I especially argue for the importance of learning in the context of treatment for anorexia nervosa and how this may relate to salient stimuli and neurotransmitter receptors. Recent studies have started to develop models how dopamine and serotonin may be related in reward learning, avoiding punishment, behavior activation as well as inhibition, suggesting opposite effects of those systems (56, 109). Problematic, however, is the complexity of especially the serotonin system with more the 14 receptor types and a lack of models how those receptors are functionally involved in behavior. There has been recent work done trying to specifically model serotonin and dopamine interaction in reward prediction, learning from punishment and how those experiences shape behavior (110), but those studies are still very theoretical. While speculative, it is important though to start a discussion of the potentially opposing functions of dopamine and serotonin in the pathophysiology and treatment in anorexia nervosa. Further, above I also speculated that the possibly serotonin driven anxious temperament and body focused anxiety in anorexia nervosa (55) may be too strong to be overcome by a dopamine related drive to eat (56), especially at the begin of the illness. With food restriction, the dopamine system could get then sensitized and its stimulation might in fact contribute to higher anxiety (101) and more food avoidance. One could then argue that stimulating the dopamine system could be counterproductive, as it would only stimulate anxiety. This is possible, yet on the other hand, if the hypothesized hypersensitive dopamine receptors are not desensitized then they would always lead to overestimation and potential food avoidance. Re-feeding probably desensitizes those receptors, but a pharmacologic intervention could accelerate that process. It is possible that in order to treat anorexia nervosa with a medication regimen one would need to target dopamine D1 and D2 receptors but possibly also the serotonin system to reduce anxiety. It is conceivable that SSRIs could be helpful to reduce anxiety, but hypersensitive dopamine receptors could trigger especially food related anxiety and defeat benefits from the SSRI medication. A similar argument could be made for cognitive flexibility and whether improved cognitive flexibility due to dopamine receptor stimulation could result in higher anxiety. These are important questions to be tested, as it is well possible and probably likely that dopamine receptor stimulation alone may not be sufficient to treat the complex state and trait related alterations in anorexia nervosa.

## Conclusion

**Figure 1:**
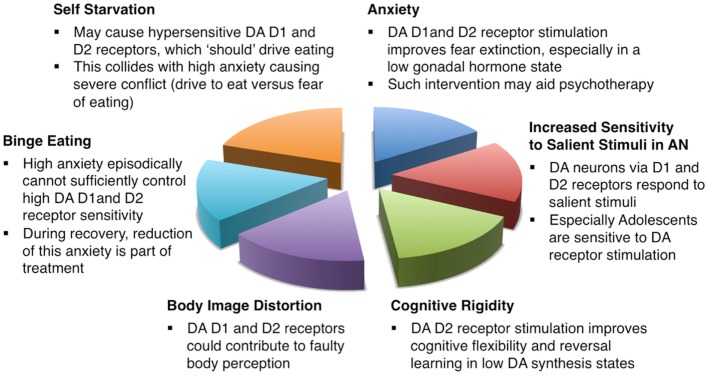
**Schematic description how dopamine D1 and D2 receptors could be involved in the pathophysiology and treatment of anorexia nervosa**.

Anorexia nervosa continues to be an incredibly difficult-to-treat disorder with high mortality and limited treatment options. This review presents a theoretical approach to this problem and provides evidence that the dopamine D1 and D2 receptors could be involved in core and associated symptoms of anorexia nervosa. Figure 1 summarizes how this neurotransmitter receptor system could be related to anorexia nervosa core behaviors. Those receptors might be particular sensitive to environmental influence such as food restriction already early in life in individuals who will develop anorexia nervosa (102). Dopamine receptor sensitivity then could be related to heightened sensitivity to salient stimuli in anorexia nervosa (51), interfere with normal fear conditioning and extinction, as well as alter food approach. Thus, a premorbid vulnerability in dopaminergic function could then become a central factor in the pathophysiology of anorexia nervosa. If such an individual is in addition highly preoccupied with the desire to lose weight perhaps because of low self esteem and high anxiety, then the combination of cognitive and fear driven behaviors together with a biological sensitivity and adaptation to food restriction might lead to the development of anorexia nervosa.

Several dopaminergic medications have been tested in treating anorexia nervosa but they were for the most part dopamine receptor antagonists, which could in fact make a hyper-sensitivity of the system worse. On the contrary, as described above dopamine receptor stimulation could reduce such receptor sensitivity and promote behavior change. It is unlikely that such medication reduces core symptoms such as drive for thinness and body image distortion “by itself” as for instance an antidepressant resolves depressed mood and anhedonia in major depression. Much rather dopamine receptor stimulation may aid in fear extinction in the context of psychotherapy and learning, as well as reduce a hypersensitive or hyperactive dopamine receptor system over time. Further, dopamine receptor manipulation might also work best in conjunction with a serotonin system specific agent, as both systems interact and are altered in anorexia nervosa. It may also be necessary to develop pharmacological strategies that are specific for the underweight state and such a regimen may then need to be adjusted when the individual with anorexia nervosa reaches normal weight and normalization of gonadal hormones.

In summary, there is evidence from basic science and some indication from human studies that dopamine D1 and D2 receptor stimulation could be a helpful pharmacological intervention for anorexia nervosa. Future research will need to test those hypotheses and also whether there is a difference in effectiveness in children and adolescents compared to adults, or whether the effects may be generalizable to the majority of anorexia nervosa patients.

## Conflict of Interest Statement

The author declares that the research was conducted in the absence of any commercial or financial relationships that could be construed as a potential conflict of interest.
